# Customized double‐shell immobilization device combined with VMAT radiation treatment of basosquamous cell carcinoma of the scalp

**DOI:** 10.1002/acm2.12536

**Published:** 2019-01-24

**Authors:** Noor Mail, Suliman M. Al‐Ghamdi, Carelse Chantel, Farid Sedhu, Atique Rana, Abdelhamid Saoudi

**Affiliations:** ^1^ King Abdullah International Medical Research Center (KAIMRC)/King Saud bin Abdulaziz University for Health Science Ministry of National Guard Health Affair Jeddah Saudi Arabia; ^2^ Princess Norah Oncology Center (PNOC) Department of Radiation Oncology King Abdulaziz Medical City Ministry of National Guard Health Affair Jeddah Saudi Arabia

**Keywords:** double‐shell‐bolus, *in vivo* dosimetry, plan quality parameters, treatment outcome

## Abstract

Malignancies with a superficial involvement of the scalp/skull present technical challenges for radiation‐treatment‐planning, such as achieving skin coverage with the prescribed dose and with the desirable conformity, homogeneity, and lower brain dose. We report a radiotherapy treatment technique for a patient diagnosed with diffuse basosquamous cell carcinoma of the scalp and adjacent skull‐bone. This study presents the plan's quality parameters, patient's dosimetry, and patient's outcome. The patient was treated using volume‐modulated‐arc therapy (VMAT) and a double‐shell‐bolus full‐head device (DSBFD) designed for patient immobilization and better skin coverage. A VMAT plan was generated using an Eclipse treatment‐planning system for a prescribed dose of 60 Gy in 30 fractions. The treatment plan was analyzed to determine the conformity index (CI), the homogeneity index (HI), the target‐coverage, and the dose to the organs‐at‐risk (OARs). Skin‐doses were measured using optically stimulated luminescence (OSL) dosimeters. Clinical follow‐up was performed by the radiation oncologist during and after the course of radiotherapy. With regard to planning target volume (PTV) coverage, the *V*_95_ was 99%. The measured and calculated dose to the skin was in the range 100–108% of the prescribed dose. The mean brain‐PTV dose was 711 cGy. The CI and HI were 1.09 and 1.08, respectively. The mean positioning accuracy for the patient over the course of treatment was within 2 mm. The measured accumulated skin dose and planning dose was agreed within 2%. Clinical examination of the patient 6 months after radiotherapy showed good response to the treatment and a 90% reduction in scarring. The DSBFD technique combined with RapidArc treatment was useful in terms of the target dose distribution and coverage. Daily patient alignment was found very precise, reproducible and less time‐consuming.

**Abbreviations**
PTV: planning target volume
CTV: clinical target volume
CI: conformity index
HI: homogeneity index
DSB: double‐shell‐bolus
CBCT: cone beam computed tomography
CT: computed tomography
VMAT: volume‐modulated arc therapy
IMRT: intensity‐modulated radiation therapy
TPS: treatment planning system
OAR: organ‐at‐risk
*V*_95_(%): the PTV volume receiving 95% of the prescribed dose
*D*_95_(%): the dose (%) received by the 95% volume of the PTV
OSLD: optically stimulated luminescence dosimeters
AAA: analytical anisotropic algorithm
HT: helical tomotherapy

## 1 INTRODUCTION

Radiotherapy is the only treatment option for basosquamous cell carcinoma of the scalp when surgery is not recommended, such as in cases of large lesions that involve the skull and brain, or owing to cosmetic and reconstructive issues, or when the patient is unwilling to undergo surgery. These extensive scalp lesions present a challenge for radiation therapy because of the convex shape of the target and its proximity to critical structures.

Traditionally, electron‐beam techniques have been used for total scalp irradiation.^1^, ^2^, ^3^, ^4^, ^5^, ^6^ Electron beams deliver high‐surface doses and exhibit rapid dose falloff, which can decrease the radiation exposure of underlying brain tissues; however, their dose distributions are inhomogeneous. Mold brachytherapy has also been widely used for the treatment of superficial skin tumors.^7^, ^8^, ^9^, ^10^, ^11^ Both brachytherapy and electron‐beam techniques are effective for superficial diseases, but may not provide suitable coverage for the infiltrated disease to the skull and brain due to the heterogeneous nature (i.e., the scalp, skull, and brain) and potentially large depth of the target.

In comparison to static and arcing electron techniques, intensity‐modulated radiation therapy (IMRT) has shown^12^ superior target coverage of the treatment volume owing to its ability to produce concave or convex dose distributions; however, this method delivers high doses to the brain and eyes. Coplanar RapidArc has been used to treat scalp disease^13^ and achieved a better dose conformity than 9‐field IMRT. Hu et al.^14^ explored the feasibility and efficiency of RapidArc in total scalp irradiation using the target‐splitting technique and noncoplanar volume‐modulated arc therapy (VMAT) planning, and suggested that the method provided a more promising solution than fixed‐beam IMRT; however, conventional RapidArc technique has shown high‐mean dose to the brain and low coverage at the skin.^14^ A dosimetric comparison between helical tomotherapy (HT) and conventional VMAT showed that HT allows the best target coverage and conformity with the lower mean dose to the brain.^15^ Most of the published articles with tomotherapy^16^, ^17^, ^18^ techniques claim good target coverage and lower‐mean dose to the brain in the scalp/skull radiotherapy treatment compared to conventional VMAT and IMRT. The linac‐based VMAT which is available almost in every cancer centers has greater potential to improve skin coverage, dose homogeneity, conformity, and reduce the mean dose of the brain with the application of 1‐cm‐thick bolus. Because the patient's skin was included in the clinical target volume (CTV), the physician aimed at the delivery of 100% of the prescribed dose to the skin; this goal was difficult to achieve with the desired dose conformity and homogeneity without the bolus application. Initially the patient's CT simulation was decided for conventional bolus but on the day of the CT simulation, there were several complexes faced and realized including (a) it was difficult to flash the bolus over the whole scalp, (b) placement reproducibility and accuracy every day treatment, (c) patient alignment issue, and (d) time‐consuming.

A double‐shell‐bolus full‐head device (DSBFD) was designed for patient‐immobilization and better coverage of the CTV especially skin. Here, we report a linac‐based radiotherapy treatment technique, VMAT, combined with the DSBFD for a patient diagnosed with diffuse basosquamous cell carcinoma of the scalp and adjacent skull bone. It is worth mentioning to report that the DSBFD device is consistently reproducible, precise, and take short time in every day placement compared to conventional bolus.

Furthermore, the skin dose must be verified with a reliable dosimeter even if 100% skin coverage is predicted by the treatment planning system (TPS). In addition, the patient's daily positioning accuracy during treatment is very important because of the tight margin created by the inclusion of skin in the planning target volume (PTV) and the close proximity of the PTV to the brain.

We present the applied VMAT‐based radiotherapy method and the design and manufacture of a head immobilization device, DSBFD, used to achieve 100% dose coverage at the skin and reduce dose to the OAR especially mean dose of the brain. Furthermore, we achieved good planning quality parameters, including the conformity index (CI), homogeneity index (HI), PTV coverage (*V*_95_, *D*_95_), and doses to the organs‐at‐risk (OARs). In addition, we provide the patient's dosimetry results, including daily and accumulated skin dose measurements with optically stimulated luminescence dosimeters (OSLDs), and discuss in detail the impact of the DSB device on target coverage and patient positioning. Finally, we report on the radiation oncologist's observations of the patient during and after radiotherapy and on the patient's outcome according to the results of the post‐treatment MRI.

## 2 MATERIALS AND METHODS

### 2.A Patient history and diagnosis

A 53‐year‐old woman presented in our department with a typical history of an old burn on her head that occurred at the age of 5 and was followed by skin grafting at that time. At the time of the examination, the patient had a nonhealing ulcer at the graft site that had lasted for 1 yr and was gradually increasing in size. Physical examination showed a large ulcer that was approximately 15 × 10 cm^2^ in size and covered almost the entire vertex [Fig. 8(a)]. Palpation of the lesion revealed that it was immobile, adherent to bone, had averted margins and granulation, and bled easily when touched. A complete head and neck examination showed no other ulcers or lesions. No neck lymph nodes were palpable. A punch biopsy showed ulcerative, moderately differentiated, keratinizing basosquamous cell carcinoma. CT of the head and neck performed prior to treatment showed focal skin thickening and ulceration of the left higher scalp region with an underlying aggressive bony lesion of the parietal bone that was consistent with the patient's squamous cell carcinoma. The metastatic workup was negative. The lesion was inoperable owing to its large size and its adherence to bone, and a complete excision with wide negative margins was not achievable in this location. As a result, the patient was scheduled for external‐beam radiotherapy with 60 Gy, to be delivered in 30 fractionations. The patient tolerated the treatment well and showed no signs of severe toxicity.

### 2.B Disease description

Marjolin's ulcer is a term used to describe a rare type of squamous cell carcinoma occurring at sites of chronic burn or scars. The malignant transformation is usually slow, with an average latency time of approximately 30 years. The tumor may initially present as an ulceration that fails to heal, and nodules may develop as the lesion progresses. Other clinical signs include rolled or averted wound margins, excessive tissue granulation, rapid increase in size, and bleeding to the touch. Squamous cell carcinomas occurring at sites of chronic burn or scars are typically aggressive and associated with a poor prognosis. Surgery remains the main treatment for Marjolin's ulcer, and wide surgical excisions with margins of up to 2–4 cm are commonly suggested. Because of their relatively poor vascularity due to extensive fibrosis, these cancers show poor response to systemic chemotherapy and radiotherapy.^19^ However, radiotherapy has been shown to be useful in the inoperable primary or recurrent tumors.

### 2.C Design and construction of immobilization device

The main objective of the immobilization device for the patient's head was to achieve 100% skin coverage and ensure an accurate and reproducible daily setup. Another aim of this design was to treat the patient in the prone position, owing to the location of the lesion. The device comprised unique anterior and posterior shells designed to cover the patient's head and secure the 1‐cm‐thick bolus flush against the target area. The design and fabrication process of the shells are described below.

#### 2.C.1 Anterior shell (mask)

**Figure 1:**
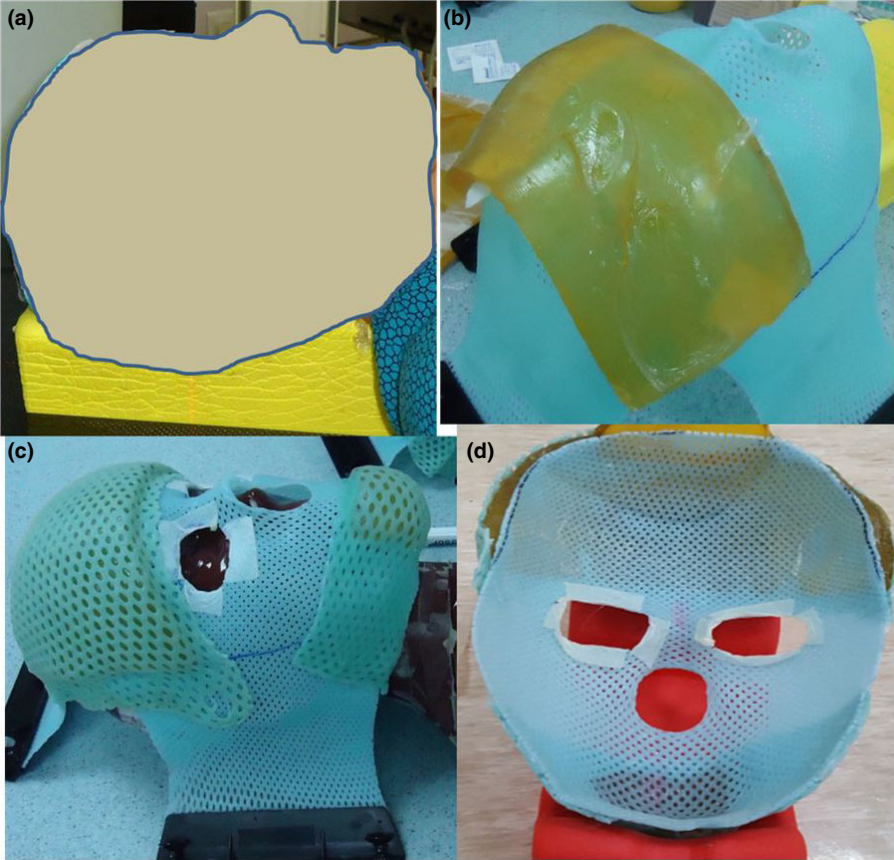
Steps for the anterior DSB device, (a) the patient was placed in the supine position on a headrest (b) Shell molded over the entire face and bolus in place at the forehead (c) another shell molded at the forehead and chin area, (d) Picture of the final anterior DSB device.

This shell was used for the immobilization of the face when the patient was in the prone position. The mask covered the face from the forehead to the chin and extended laterally to the level of the ears (Fig. 1).

- The patient was placed in the supine position on a comfortable headrest that was selected to fit her head and neck as shown in Fig. 1.
- Next, the patient was aligned in the treatment position using anatomical landmarks, such as the tragus, septum, brows, and superior sternal notch. Anterior scout images were acquired using a CT scanner to confirm the position. The midline was marked to guide the therapist during the shell construction process and ensure the patient's alignment was maintained.
- A three‐point shell was placed in a hot water bath until translucent. Subsequently, the shell was patted dry and stretched over the patient's face. A good molding over the entire face was ensured and special care was given to the forehead, nose, chin, and cheeks. Bolus was sandwiched between the shells at the forehead area.
- After the mask was set, it was removed from the patient.
- The mask was cut to include the entire face area to the level of the ears.

#### 2.C.2 Posterior shell

This shell was designed for the immobilization of the posterior aspect of the head and neck area and was used as a repositioning guide during treatment.

**Figure 2:**
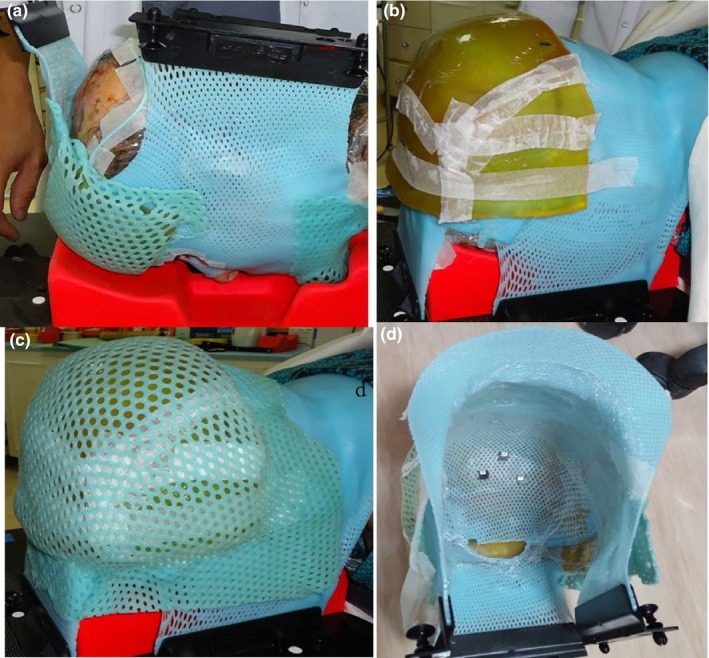
Steps for the posterior DSB device, (a) Picture of the posterior inner shell, (b) Bolus was in placed over the posterior inner shell, (c) Outer shell was placed over the bolus, (d) Picture of the final posterior DSB with three OSLDs in placed.
- A posterior scout image was obtained to verify the patient's alignment.
- The anterior mask was fixed to the prone headrest (a glue gun was used).
- A three‐point shell was placed in a hot water bath until translucent. The shell was then patted dry and stretched over the patient's posterior head and neck area. A good molding of the shell over the neck and skull area was ensured.
- When the posterior shell was set and completely dried, the bolus was added. The thickness and area of the bolus were determined by the radiation oncologist and verified by the medical physicist. During application, a better attachment to the shell was achieved by removing the skin of the bolus and placing its sticky side facing the shell. We ensured that there were no gaps or overlap resulting from the irregular shape of the area to be covered.
- A thermoplastic sheet was placed in a hot water bath. Next, the sheet was positioned over the bolus and attached to the posterior shell (the bolus was permanently sandwiched between the shells). As advised by the medical physicist and the radiation oncologist, the therapist performed a CT scan of the patient in the treatment position (see Section 2.D).

- The anterior shell was placed on the prone headrest. The patient was then placed in a prone position with her arms by her sides. A mattress and an ankle rest placed under her ankles were used for comfort. The posterior shell is shown in Fig. 2.

### 2.D CT scanning and simulations

CT simulation of the patient is required for radiation dose calculations. The patient was immobilized using the DSB device. The patient was scanned in the prone position using a Siemens CT scanner (Somatom Sensation Open, Siemens Medical Solutions, Erlangen, Germany) under the standard brain imaging protocol (120 kVp and 120 mAs). Images were reconstructed with a voxel size of 1 × 1 × 2 mm^3^.

### 2.E RapidArc treatment planning and delivery

The delineation of the CTV and the PTV was performed by the radiation oncologist using the Eclipse TPS (version 11.0, Varian Medical Systems, Palo Alto, CA, USA). For the CTV‐to‐PTV expansion, a 3 mm margin was applied. The OARs, including the eyes, lenses, optic nerves, optic chiasm, and brain‐PTV, were delineated by the medical physicist and reviewed by radiation oncologists.

**Figure 3:**
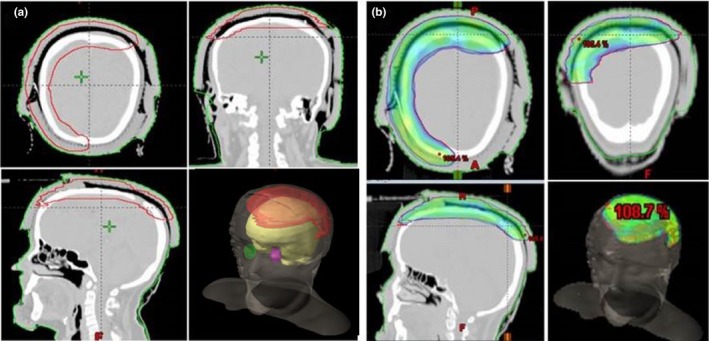
(a) The planning target volume and (b) 95% prescribed dose distribution. The prescribed dose was 6000 cGy at 200 cGy/fraction. The objective was to achieve 100% coverage at the skin.

The CT images and OAR contours are shown in Fig. 3(a). The prescribed dose to a single PTV was 6000 cGy. The normal tissue objective option was used for better dose shaping and the sparing of normal tissue. Higher priorities were given to the PTV coverage and normal tissues sparing especially brain. The final dose calculation was computed with the analytical anisotropic algorithm (AAA) version 11.0 and a grid voxel size of 2.5 × 2.5 × 2.5 mm^3^. A high‐skin dose within the PTV was among the highest priorities. The objective was to achieve more than 100% of the prescribed dose at the skin and reduce the dose to the OAR. The RapidArc treatment was delivered using two arcs (clockwise and counterclockwise) from a 6 MV beam with a single isocenter, 350° gantry rotation, collimator angles of 15° and 345° and a maximum dose rate of 600 MU/min.

### 2.F Plan evaluation

The approved treatment plan was evaluated according to standard planning quality parameters, including the CI, HI, dose‐volume histograms (DVHs), the mean dose to the skin in the PTV, *V*_95_, *D*_95_, the mean brain dose and doses to the OARs (eyes, lenses, optic nerves, optic chiasm, and brain). Dose fall of characteristic beyond the PTV extending into normal tissue was evaluated from the DVH curve of the Brain‐PTV structure. For the dose fall off trend, the parameters including *D*_5%_ and *D*_20%_ for the Brain‐PTV structure were closely looked by the RO during the plan approval.

#### 2.F.1 Conformity index (CI)

The CI of the treatment plan was defined using the following equation^13^:

$$\text{CI}=\frac{V_{\mathrm{PTV}}\times V_{\mathrm{TV}}}{{\text{TV}}_{\mathrm{PV}}^{2}}$$

where *V*_PTV_ is the treatment volume of the identified isodose, *V*_TV_ is the volume of the PTV, and TV_PV_ is the volume of the PTV inside the identified isodose.

#### 2.F.2 Homogeneity index (HI)

The HI was defined using the following formula:

$$\text{HI}=\frac{D_{2\%}-D_{98\%}}{D_{50\%}}\times 100$$

where *D*_98%_, *D*_50%_, and *D*_2%_ are the doses received by 98%, 50%, and 2% of the PTV volume, respectively.

### 2.G Patient alignment/positioning

For superficial and complex targets, the use of CBCT and kV imaging were highly recommended to ensure patient alignment. During treatment, an on‐board imaging (OBI) system was used to align the patient for the delivery of each radiotherapy fraction. The OBI 20 device is capable of acquiring two‐dimensional kV and three‐dimensional volume CBCT images. CBCT scan acquired once in a week and orthogonal kV images were acquired four times in a week. The CBCT images of the head and neck region were acquired with a full‐fan bowtie filter, a 2.5 mm slice thickness and a pixel size of 0.65 × 0.65 mm^2^. The CBCT exposure parameters were 80 kVp, 25 mA, and 8 ms. The setup error was determined by the anatomical displacement between the kV or CBCT images and the reference planning CT images and was expressed as translational couch shifts in three directions.

### 2.H Patient dosimetry with OSLDs

As the disease was most severe and intense at the skin of the patient, skin dose monitoring was very important. *In vivo* dosimetry for radiotherapy patients is necessary to verify that the dose delivered to the patient corresponds to the prescribed dose calculated by the TPS. The use of OSLDs is highly recommended for skin dose measurements at superficial lesions of the scalp or adjacent regions of the skull. We used a commercially available OSLD system (Landauer, Inc., Glenwood, IL, USA) that consisted of a small microStar^®^ reader and InLight^®^ nanoDot^™^ dosimeters (Al2O_3_:C), for more detail on OSLD read Mail et al.^21^ Three OSLDs were taped on the inner surface of the DSB device and positioned accurately at the tumor site, at fixed locations called A, B, and C within the CTV. OSLD‐A was read and replaced with a new annealed dosimeter daily after each fraction; OSLD‐B was read after 2–4 accumulated fractions and OSLD‐C after 30 accumulated fractions (i.e., after completion of the treatment). OSLD reads both therapeutic and imaging doses, but imaging doses from CBCT and orthogonal kV techniques were ignored because of its low magnitude in comparison to prescribed therapeutic dose.

## 3 RESULTS

### 3.A Evaluation of RapidArc treatment plan

#### 3.A.1 PTV coverage

**Figure 4:**
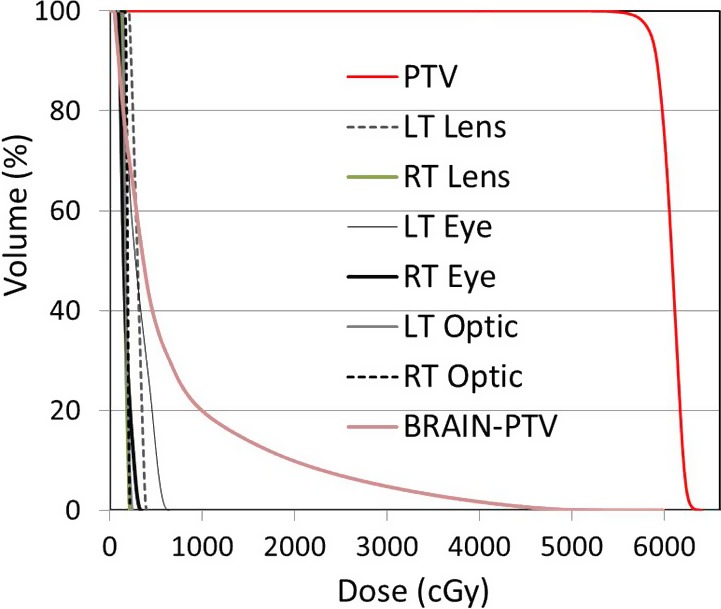
Dose‐volume histogram showing the coverage of the planning target volume and the doses to the organ at risks.

**Figure 5:**
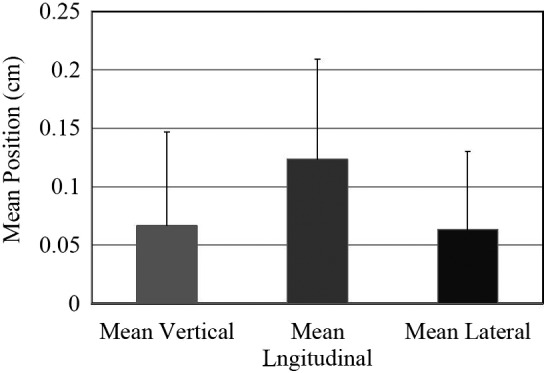
Mean positioning accuracy of the patients alignment in all three directions including vertical, longitudinal and lateral.

The 95% of the prescribed dose distribution in colorshad is shown in Fig. 3(b). The conformity and homogeneity indices for the VMAT plan were 1.09 and 1.08, respectively. Quantitative evaluations were performed using cumulative DVH analysis (Fig. 4). The dose was received by 1% volume of the PTV (*D*_1%_) values of 6240 cGy (104%). The *V*_95_ was 99% and the PTV mean dose was 6075 cGy. These dosimetric parameters show that the dose distribution within the PTV was very uniform, homogenous, and conformal. The mean skin dose measured within the PTV was 6239 cGy. From the brain‐PTV curve (Fig. 5), the dose as a function of volume reduces sharply; this is a clear indication of the sharp dose reduction from the edge of the target to the normal tissues. The parameters including *D*_5%_ and *D*_20%_ for the Brain‐PTV structure were 2946 and 993 cGy, respectively. The good dosimetric outcome is attributed to the DSB device because the optimizer software required less effort to cover the skin. The application and implementation of the 1‐cm‐thick bolus DSB reduced the mean brain dose and improved the dose distribution within the PTV while keeping the mean brain dose reasonably lower.

#### 3.A.2 Doses to OARs

The plan quality parameters including PTV, OAR, and normal tissues are listed in Table 1.

**Table 1:** Dosimetric results of the RapidArc treatment plan

Structures	Dosimetry	Results
PTV	*V*_95_ (%)	99
	*D*_95_ (%)	98
	CI	1.09
	HI	1.08
Brain	*D*_mean_	1217 cGy
Brain‐PTV	*D*_mean_	711 cGy
	*D*_5%_	2946 cGy
	*D*_20%_	993 cGy
Lens left	*D*_max_	400 cGy
Lens right	*D*_max_	217 cGy
Eye left	*D*_max_	647 cGy
Eye right	*D*_max_	342 cGy
Optic nerve left	*D*_max_	307 cGy
Optic nerve right	*D*_max_	220 cGy
Chiasm	*D*_max_	253 cGy

The maximum doses to the left and right eye were 647 and 342 cGy, respectively. The maximum doses to the left lens, right lens, right optic nerve, and left optic nerve were 400, 217, 220, and 307 cGy, respectively. The RapidArc plans with the application of the DSB technique better protected the OARs because of the easy PTV targeting without skin sparing. Our results demonstrated that all the analyzed doses to the optical structures were well‐below the tolerances established by the qualitative analysis of normal tissue effects in the clinic (QUANTEC) criteria. The mean doses to the brain and brain‐PTV were 1217 and 711 cGy, respectively. The other dosimetric parameters of the brain, *D*_50_, *D*_30_, and *D*_20_ were 414, 899, and 1899 cGy, respectively.

### 3.B Patient alignment/positioning

During the kV and CBCT imaging, the shifts and patient contours were monitored for any significant changes. The overall alignment accuracy (Fig. 5) for the vertical, longitudinal, and lateral directions was 0.7 ± 0.7, 1.23 ± 0.85, and 0.63 ± 0.65 mm, respectively. To facilitate comparisons between the three directions, the longitudinal shift was longer than both the lateral and vertical shifts. To compare CBCT and kV techniques, the mean vertical and lateral shifts were almost the same for both techniques. However, the mean shift along the longitudinal direction was smaller with CBCT (0.7 ± 0.71 mm) compared to kV images (1.4 ± 0.73 mm). The average patient's setup time with DSBFD was recorded 3.2 ± 0.5 min, which was slightly higher than the average time (3 ± 0.5 min) with conventional three‐point shells.

### 3.C Patient dosimetry with OSLD

**Figure 6:**
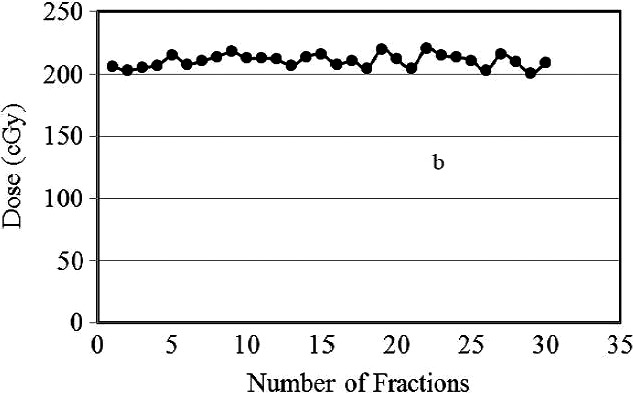
The dose measured with OSLD‐A for each fraction is plotted against number of fractions.

**Figure 7:**
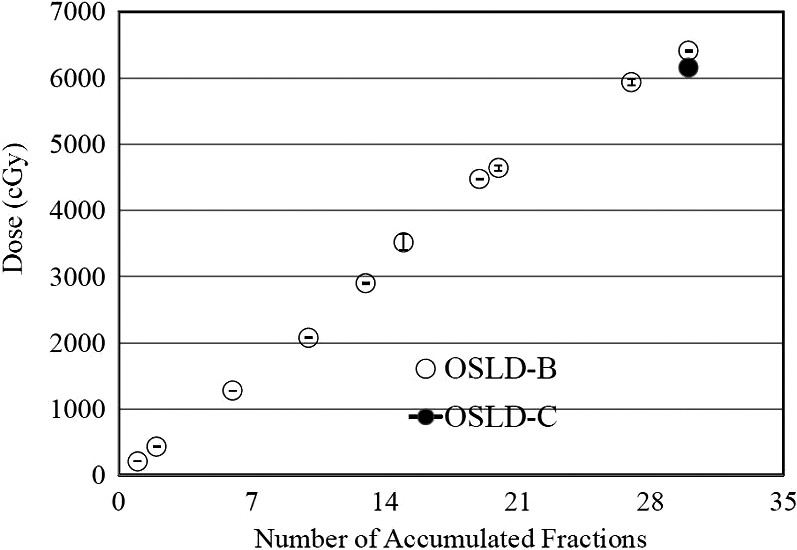
The accumulated dose measured with OSLD‐B (read after each ~3 fractions) and total accumulated dose measured with OSLD‐C (read once after the treatment completion) plotted as a function of number of fractions.

The OSLD‐A reading obtained after each fractionation is plotted versus the number of fractions in Fig. 6. The average measured skin dose for location A was 204 ± 4.4 cGy per fraction. The plot shows that the skin dose at location A measured throughout the treatment was higher than the prescribed dose by ~2%. Since, the tumor was at the skin, the objective was to irradiate the skin with 100% prescribed dose. The reading of the OSLD‐B is plotted against the number of fractions in Fig. 7. As explained in the method section that the idea of OSLD‐B was to look at the accumulated dose of 2–4 fractions that is to verify the dose per fraction measured with the OSLD‐A is accurate. The measured dose with OSLD‐B shows almost linear increase with the number of fractions. However, the data points at fraction 14 and 21 slightly deviate from the rest of the data. Each accumulated reading of the OSLD‐B was within ~2% agreement with the measured dose of OSLD‐A. The minor dose variations shown in the plot can be attributed primarily to the number of the readings (every 3 fractions). The OSLD‐B reading after the last fraction was 6390 *± *51 cGy (106% of the prescribed dose). The accumulated dose measured with OSLD‐C after 30 fractions was 6158 ± 101 cGy (102.5% of the prescribed dose), which shows the closest agreement (2%) with the dose of 6239 cGy calculated by the treatment plan at the location of OSLD‐C. The results of in vivo dosimetry for radiotherapy patients, especially for the delivered skin dose, must correspond to the prescribed dose calculated by the TPS. Typically, calculations and measurements of the skin/surface doses are always difficult and complicated; however, the calculated and measured doses obtained in this study were in close agreement.

### 3.D Patient outcome

**Figure 8:**
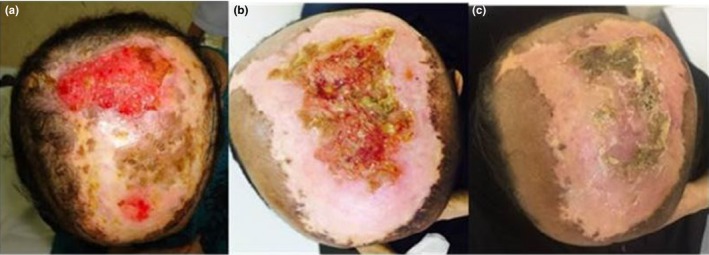
Photographs of the patient's head after diagnosis with basosquamous cell carcinoma: (a) before radiotherapy, (b) 4 weeks after radiotherapy and (c) 6 months after radiotherapy.

An MRI of the head and neck performed at 8 weeks post‐radiotherapy showed evidence of scarring and loss of subcutaneous tissue in the left front parietal region. Upon clinical examination, the patient's response to the radiation treatment was shown to be excellent, approximately 20% of the initial scarring. The scaring comparison can be seen from Fig. 8(a) and 8(b) acquired before and after the treatment, respectively. After 6 months, the patient was examined in our clinic, and the lesion was almost resolved with limited skin scarring. Figure 8(c) shows a photograph of the patient's head obtained 6 months after radiotherapy and clearly displays an improvement in the affected area in comparison with the lesion before radiotherapy.

## 4 DISCUSSION

The main goal of this study was to provide a uniform dose (with the desired skin coverage) to the target (scalp/skull) while maintaining the dose delivered to the normal tissues as low as possible (compared to the published data^13^, ^14^, ^15^, ^16^, ^17^, ^18^) using linac based VMAT technique. This therapeutic goal cannot be easily achieved without the use of the DSB device because of the concave shape of the target, lesion at the skin (the objective was 100% coverage of the skin), and the doses to the OARs. The double shell concept was to ensure comfort to the patient and more importantly to ensure daily reproducibility by immobilizing the patient in a fix position. Besides this, the role of both the anterior and posterior shell was to ensure that the bolus is properly in place and covers the treatment area as marked by the radiation oncologist. Some area of the forehead was included in the PTV, therefore the bolus in the anterior shell was adequate to use in the forehead area only. The rest of the anterior shell was in contact with the patient face without bolus. The anterior shell also acts as a customized headrest that follows the patient's facial contour. This ensures comfort to the patient as it is customized and molded to the patient's specific anatomy. The posterior double‐shell bolus is fixing the patient to the baseplate onto the Treatment coach. With the use of DSB technique, we achieved good dose homogeneity and conformity of the PTV and lower doses to the OAR, especially mean dose of the brain compared to the published data.^13^, ^14^, ^15^, ^16^, ^17^, ^18^, ^19^, ^22^, ^23^ Without the DSB application, the PTV can definitely exceed the patient's skin outline due to tumor invasion at the skin. In this case, the CTV was clearly touching the skin. The PTV margin was adequate to tolerate an alignment accuracy of 2–3 mm for patients with brain involvement. The superficial part of the PTV may extend into the buildup region of incoming photon beams or the surrounding air without bolus application. Most of the dose computation algorithms cannot accurately compute dose in the buildup regions, thus convergence errors occur when these algorithms are used for optimization.^13^ Optimizing the physical dose in air is irrelevant, but the part of the PTV outside the skin contour is not irrelevant because it should ensure the sufficient coverage of the surrounding air to prevent the CTV from reaching outside the beam edge due to deformation, movements or setup error. In RapidArc plan optimization, the role of the DSB device was very useful for solving the following critical issues: (a) CTV stops exactly at the skin surface, whilst the margin for geometric uncertainties may take the PTV beyond the skin. In these cases, optimization leads to over‐dosing the buildup and air part of the PTV to cover with the prescribed dose. This issue was resolved with the use of custom‐made DSB device. (b) Relaxing the planning optimizer for target coverage will improve dose conformity and homogeneity in the PTV and lower dose to the brain. (c) Consistent bolus location for all fractionations improved dose accuracy at the skin (which is part of the CTV), the DSB prevents target miss due to setup error (PTV can be extended to the DSB). The fabrication of the DSB device needs approximately 90 min, but it depends upon the skill and experience of the radiotherapists and physicists.

One of the major factors affecting the accuracy of treatment is patient setup error; therefore, the use of an immobilization device is imperative. The immobilization device must provide adequate rigidity to ensure maximum immobilization, the alignment accuracy of our immobilization device was measured to be within 2 mm throughout the treatment.

The doses at the skin lesion measured with OSLDs, including daily fractions and total accumulated fractions, was in good agreement with the calculated dose. The daily dose measurements at the skin lesion throughout the 30 fractions showed more than 100% of the prescribed dose. The agreement between the total measured accumulated dose and the calculated planning dose was within 2%. The daily patient dosimetry resulted in greater confidence that the skin lesion was properly aligned and received the prescribed dose. It is worthy to mention that the CBCT doses (approximately 0.35% of the prescribed dose) were ignored because of its low magnitude compared to the prescribed therapeutic dose. The patient outcome, including lesion resolution and disease control, was reasonably good. The difference between the lesion/tumor size before and after radiotherapy can be observed from the pre‐ and post‐radiotherapy images in Fig. 8(a)‐(c).

## 5 CONCLUSIONS

The use of the DSB device was determined to significantly aid with the coverage of a complicated (concave) shape and a superficial target (skin lesion). Our objective of 100% coverage of the skin was successfully achieved. Furthermore, the implementation of the DSB improved dose homogeneity and conformity in the PTV and showed reduction in mean dose to the brain compared to the published data.^13^, ^14^, ^15^, ^16^, ^17^, ^18^, ^19^, ^22^, ^23^ The patient alignment accuracy in all three directions was within 2 mm throughout the treatment.

The dose measurements at the skin lesion with OSLDs, including daily and total accumulated doses, were useful for determining dose accuracy and the level of agreement with the planned dose. Also, it gives a strong clue regarding the patient alignment throughout the treatment duration in the form of dose accuracy at the skin. The daily dose measurements at the skin lesion were achieved 100% of the prescribed dose throughout the treatment. The agreement between the measured dose and the planning dose was within 2%.

## 6 DECLARATIONS

### Ethics approval and consent to participate

This study was reviewed and approved by the Research Ethics Boards (Institutional Board Review) of the King Abdullah International Medical Research Center (KAIMRC), Ministry of National Guard Health Affairs, Saudi Arabia. Consent of participant in this study was obtained from the patient.

### Consent for publication

Available on request.

### Competing interests

The authors declare that they have no competing interests.

### Funding

No funding has been used or requested.
